# Strategy modulates spatial perspective-taking: evidence for dissociable disembodied and embodied routes

**DOI:** 10.3389/fnhum.2013.00457

**Published:** 2013-08-13

**Authors:** Mark R. Gardner, Mark Brazier, Caroline J. Edmonds, Petra C. Gronholm

**Affiliations:** ^1^Department of Psychology, University of WestminsterLondon, UK; ^2^School of Psychology, University of East LondonLondon, UK; ^3^Health Service and Population Research Department, Institute of Psychiatry, King’s College LondonLondon, UK

**Keywords:** perspective-taking, own body transformation, strategy, embodiment, social, response inhibition

## Abstract

Previous research provides evidence for a dissociable embodied route to spatial perspective-taking that is under strategic control. The present experiment investigated further the influence of strategy on spatial perspective-taking by assessing whether participants may also elect to employ a separable “disembodied” route loading on inhibitory control mechanisms. Participants (*N* = 92) undertook both the “own body transformation” (OBT) perspective-taking task, requiring speeded spatial judgments made from the perspective of an observed figure, and a control task measuring ability to inhibit spatially compatible responses in the absence of a figure. Perspective-taking performance was found to be related to performance on the response inhibition control task, in that participants who tended to take longer to adopt a new perspective also tended to show a greater elevation in response times when inhibiting spatially compatible responses. This relationship was restricted to those participants reporting that they adopted the perspective of another by reversing left and right whenever confronted with a front-view figure; it was absent in those participants who reported perspective-taking by mentally transforming their spatial orientation to align with that of the figure. Combined with previously published results, these findings complete a double dissociation between embodied and disembodied routes to spatial perspective-taking, implying that spatial perspective-taking is subject to modulation by strategy, and suggesting that embodied routes to perspective-taking may place minimal demands on domain general executive functions.

## Introduction

Spatial perspective-taking underlies successful social interactions (Tversky and Hard, 2009), for instance when giving directions or demonstrating how to perform a task. Furthermore, spatial perspective-taking itself may be an intrinsically social process, when the novel perspective one adopts is that occupied by another person, rather than a position external to that occupied by any other body (Stocker, 2012). Although it has been well established that qualitatively different underlying processes subserve different kinds of perspective-taking (e.g., Michelon and Zacks, 2006; David et al., 2008; Cohen et al., 2009), the manner in which these various perspective-taking mechanisms rely on “embodied” cognitions such as the mental simulation of body movements has yet to be fully specified—despite this being an active line of enquiry (e.g., Kessler and Rutherford, 2010; Kessler and Thomson, 2010). Investigation of embodied perspective-taking may help to elucidate how the spatial and social domains impinge upon perspective-taking. Outstanding issues include identifying the types of perspective-taking that are possible via a “disembodied” route that engages response inhibition rather than motor simulation or social processes, as well as the role played by endogenous control processes in selecting between multiple perspective-taking routes. Consequently, the aim of the current study was to examine how these two types of executive processes influence perspective-taking, by assessing whether the strategy that participants report using moderates the relationship between perspective-taking ability in the “own body transformation” (OBT) task (e.g., Zacks et al., 1999; Blanke et al., 2005; Mohr et al., 2010) and ability to perform a control task indexing disembodied response inhibition processes.

The prevailing view is that spatial perspective-taking via imagined transformations of one’s own egocentric perspective is an embodied process (e.g., Kessler and Rutherford, 2010; Kessler and Thomson, 2010), in the sense that it is performed via mental simulation of the sensorimotor mechanisms involved in actual self rotation (Lenggenhager et al., 2008). The finding that the speed and accuracy of taking another’s viewpoint depends upon the degree of angular disparity between one’s own and a target’s frame of reference provides evidence for an analogue transformational process sharing at least some of the properties of self motion (Zacks and Michelon, 2005). Support for the involvement of deliberate motor simulation is provided by reports that postural congruence between participants and targets facilitates perspective-taking performance (Kessler and Rutherford, 2010; Kessler and Thomson, 2010). In addition, an individual’s motor capability appears to modulate the extent that motor simulation is engaged in perspective-taking. For instance, skill at performing rotational movements has been found to facilitate perspective-taking during a mental body rotation task (Steggemann et al., 2011), and attentional biases associated with participants’ own handedness have been found to extend to left-right judgments made from a schematic figure’s perspective in the OBT task (Gardner and Potts, 2010). Furthermore, patients with left spatial neglect have even been found to recover information that is unavailable from an egocentric perspective when space is imagined from an opposite perspective (Becchio et al., 2013). Of particular relevance, the degree of amelioration of the neglected side is greatest in an embodied condition when a person is seen to be present in the novel perspective. These findings provide converging evidence for embodied processes contributing to perspective-taking.

Nonetheless, under certain circumstances disembodied processes appear sufficient to account for perspective-taking. For instance, determining which objects can be *seen* from another person’s perspective appears to involve line-of-sight computation without the need for transformations of one’s own perspective (Michelon and Zacks, 2006). The determination of spatial relationships relative to a third party perspective within the OBT task has also been accounted for in terms of domain general response selection processes and spatial compatibility, either alone (Gardner and Potts, 2011), or in combination with imagined perspective transformations (May and Wendt, 2012). In these cases, a conflict arises between information coded relative to one’s own bodily position and information coded for the adopted perspective (May, 2004; Michelon and Zacks, 2006). Thus, the cognitive demands of perspective-taking are at least in part due to the need to inhibit prepotent responses relating to one’s own perspective (cf Leslie et al., 2005). In support of this view, the ability to adopt a third party perspective has been shown to be disrupted when performed alongside a secondary task loading on response inhibition processes (Qureshi et al., 2010). Thus, “disembodied” executive functions, including response inhibition, may contribute to perspective-taking alongside, or in place of, a cognitively efficient “embodied” route.

One possibility is that separable embodied and disembodied perspective-taking processes (May and Wendt, 2012), may in fact be distinct routes to perspective-taking controlled by higher level strategy (Gronholm et al., 2012). Although many have proposed that utilization of different strategies could explain variation in perspective-taking performance (Michelon and Zacks, 2006; Thakkar et al., 2009; Mohr et al., 2010; Thakkar and Park, 2010), the role of strategy has rarely been considered explicitly (Amorim, 2003). Initial evidence has been reported indicating the presence of a dissociable strategy associated with embodied perspective-taking (Lenggenhager et al., 2008; Gronholm et al., 2012). For instance, the disruption to mental transformations arising from galvanic vestibular stimulation was found to be restricted to participants reporting that they had employed transformations of their own perspective, rather than an object based strategy (Lenggenhager et al., 2008). Using the OBT task, Gronholm et al. (2012) found a selective association between trait level empathy and perspective-taking ability that was restricted to participants using an embodied perspective transformation strategy, as opposed to disembodied strategy of reversing left and right whenever confronted with a front-view figure (Gronholm et al., 2012). This finding is consistent with mental simulation playing a common role for embodied spatial perspective-taking as well as social processes such as empathy and Theory of Mind (Ruby and Decety, 2004). However, to date, no equivalent independent evidence appears to be available for disembodied perspective-taking strategies.

The current study was designed to assess further the influence of strategy on perspective-taking in the OBT-task, by examining whether the strategy that participants report using moderates the relationship between perspective-taking and response inhibition abilities. Previous work using an individual differences approach has found that perspective taking is associated with response inhibition ability (Qureshi, 2008). Here, we examine whether this association is strategy-specific. In the present study, participants undertook both the OBT perspective-taking task, requiring speeded spatial judgments made from the perspective of an observed figure, and the “Transpose” task, a disembodied control task measuring ability to inhibit spatially compatible responses. We predicted that if there are dissociable embodied and disembodied routes to spatial perspective-taking that are modulated by high level strategy, then self-reported strategy should moderate the relationship between performance on the OBT and Transpose tasks. Specifically, we predicted a positive relationship between performance for the OBT and Transpose tasks that would be restricted to those who reported that they adopted the perspective of another by transposing left and right whenever confronted with a front-view figure; no association was predicted for those who in an embodied manner mentally transformed their perspective to align with that of the external figure.

## Materials and methods

### Participants

Ninety two volunteers (69 female, 23 male), recruited primarily from the university community, took part in the study. Their ages ranged from 19 to 66 years (mean ± SD = 24.4 ± 9.4 years). All had normal, or corrected to normal, vision, and provided informed consent in accordance with the local (University of Westminster) ethics approval.

### OBT task

**Figure 1:**
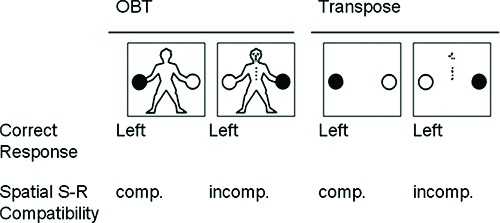
**Schematic illustration of the relations between stimuli and responses for both the OBT and Transpose tasks.** For both tasks, stimulus-response (S-R) mappings were compatible for 50% of the trials (back-view; cue absent), and incompatible for the remainder (front-view; cue present).

The OBT task was adapted from that reported previously (Gardner and Potts, 2011, Experiment 1A), as summarized below. Four basic stimuli each depicting a schematic human figure holding a black ball in one hand and a white ball in the other, were presented to participants. The figure could be seen either from front- or back-view, and held the black ball in either the left or right hand (see Figure 1, illustrating left hand stimuli). The outline shape of the figure was identical whether it was front- or back-facing. Consequently, the only aspects of the stimulus indicating that the figure was front-facing were the marks indicating the buttons and facial features.

Participants were verbally instructed to imagine taking the perspective of the figure through an embodied mental transformation in order to make a spatial judgment as to which hand the figure was holding the black ball. Standardized instructions to this effect were also delivered via the E-prime program. Each participant was required to rest their index fingers on the response keys (left index finger on the “A” key for a “left” response on a QWERTY keyboard, and right finger on the “L” key for a “right” response). This resulted in S-R mappings that were spatially compatible for 50% of the trials (back-view) and spatially incompatible for the remainder (front-view; see Figure 1).

### Transpose task

The Transpose task (Gardner and Potts, 2011) served as a disembodied control task measuring ability to inhibit spatially compatible responses. The stimuli consisted of two balls, one black and one white, in identical locations to those appearing in the OBT task, but in this case presented without a human figure holding them. The black ball could appear on the left or the right, and was presented either alone, in the cue-absent condition, or accompanied by an abstract visual cue, in the cue-present condition. This abstract visual cue consisted of the features that made up the OBT figure’s face and buttons presented in a scrambled configuration. Thus, these stimuli served as non-embodied variants of those employed for the OBT task (see Figure 1).

Participants were instructed to report the location of the black ball from their own viewing perspective by pressing the corresponding key when the abstract visual cue was absent. On trials in which the cue was present, participants were required to transpose left and right when responding (e.g., if the black ball was on the right, the correct response was to press the left response key). Thus, just as in the OBT task, the mapping between stimulus location and response was spatially incompatible for 50% of the trials, and these trials were signaled by equivalent visual information—those marks distinguishing front- and back-view stimuli in the OBT task. The Transpose task should thus place similar demands on response inhibition processes as the OBT task for those participants adopting a transposing strategy, given that it is operationalized in a comparable manner.

### Procedure

All participants performed the OBT task, followed by the Transpose task. The order of these tasks was not counterbalanced in order to prevent expected carry-over to the strategy adopted for the OBT task if participants had experienced the Transpose task first. On each trial, a central black fixation cross was presented for 1400 ms against a white background. This was immediately followed by the stimulus which was displayed for 2100 ms, or until a response had been made. This was followed by visual feedback on whether the response was correct or incorrect, presented for 1500 ms. On any given trial, the stimulus was randomly displaced in the picture plane (range of −50° to +50°, in 10° intervals) to introduce further variability in the stimulus set. Each task comprised 132 trials split into two equal blocks, allowing all stimulus combinations to be presented on three occasions in a random order [left vs. right (2) x compatible vs. incompatible (2) x picture plane orientation (11)]. Stimulus presentation and data collection were controlled by a personal computer running E-Prime experiment generator software (Schneider et al., 2002).

Immediately after these tasks were completed participants were asked to report on the strategy they had used during the OBT task, based on which they were categorized into “perspective transformers” or “spatial transposers” in accordance with earlier work (Gronholm et al., 2012). This was intended to discriminate strategies on the basis of embodiment. Those who reported to have always/usually used the “flipping left and right strategy” were classified as (disembodied) spatial transposers, whereas those who always/usually “imagined myself taking the figure’s position” were classified as (embodied) perspective transformers. Participants were classified as perspective transformers also if they reported having used both strategies equally often.

## Results

Participants were excluded from the analysis due to an error rate (ER) of above 15% on either the Transpose task (*N* = 5, all female), or the OBT task (*N* = 14, 12 female). The sample that was subjected to analysis thus comprised 73 participants (51 female). In order to measure the relative increase in response times for the incompatible versus compatible condition, a “Composite response time (RT)” for both tasks was computed for each participant according to the formula: Composite RT = (incompatible RT—compatible RT)/compatible RT—see Gronholm et al. (2012). Shapiro-Wilks test indicated these data to be normally distributed: OBT task, *W* = 0.973, *p* = .124; Transpose tasks, *W* = 0.989, *p* = .793.

### Reported strategy use, and performance on perspective-taking and response inhibition tasks

According to self-report, for the OBT task 43 participants (59%) adopted the disembodied transposing strategy and 29 (40%) adopted the putatively embodied perspective transformation strategy. Data on strategy use was unavailable for one further participant. The difference between these proportions was not statistically significant, *p* = .125, binomial test. By adopting the same classification criterion as Gronholm et al. (2012), the embodied perspective transformation subgroup included 11 participants (38%) that reported having used both strategies. The strategy subgroups were not found to differ in terms of gender distribution, χ^2^ = 1.69, *p* = .194, nor age, *t*(69) = 0.01, *p* = .992 (embodied perspective transformation: 79% female, age (mean ± SD) = 24 ± 9.2 yrs; disembodied transposing strategy: 65% female, age = 24 ± 9.5 yrs).

**Figure 2:**
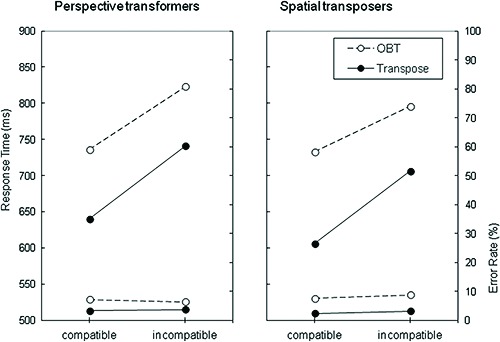
**Mean of correct response times in ms and error rate (%) for both the OBT and Transpose tasks as a function of strategy reported (perspective transformers vs. spatial transposers) and compatibility (compatible vs. incompatible)**.

Figure 2 illustrates RT and ER performance on both the OBT and Transpose tasks categorized by the strategy reported, and the S-R compatibility of the stimuli. RTs appeared to be longer for the OBT task than the Transpose task, and longer for the incompatible condition relative to the compatible condition, irrespective of the strategy reported. These impressions were confirmed by a 3-way mixed model Analysis of Variance (ANOVA) where Task (OBT vs. Transpose) and Compatibility (compatible vs. incompatible) were within subject factors and Strategy (perspective transformers vs. spatial transposers) was a between subject factor.This revealed main effects of Compatibility, *F*(1,70) = 161, *p* < .001 and Task, *F*(1,70) = 70.1, *p* < .001, neither of which interacted with Strategy, *F*s < 1. Furthermore, the main effect of Strategy was not significant, *F* < 1. An interaction between Task and Compatibility was found, *F*(1,70) = 5.48, *p* < .022, consistent with a higher elevation of response times for the incompatible relative to the compatible condition in the Transpose task (mean ± SD = 17 ± 12%) than in the OBT task (11 ± 11%). This phenomenon also did not interact with Strategy, *F*(1,70) = 1.017, *p* = .317.

An equivalent 3-way ANOVA was also performed on the ER data depicted in Figure 2. This revealed that ER was higher for the OBT task (mean ± SD = 7.5 ± 4.1%) than the Transpose task (3.1 ± 2.8%), *F*(1,70) = 68.2, *p* < .001. However neither the main effect for Compatibility, *F* < 1, nor that for Strategy, *F* < 1, were statistically significant. Strategy was found to moderate the size of the Task effect, *F*(1,70) = 4.04, *p* < .048. The degree to which participants showed greater accuracy for the Transpose compared with OBT task was slightly greater for those reporting having adopted the disembodied spatial transposing strategy (difference in ER, mean ± SD = 5.4 ± 4.4%, *t*(42) = 8.1, *p* < .001), than for those reporting having adopting a perspective transformation strategy, (3.3 ± 4.4%, *t*(28) = 4.0, *p* < .001). No other interactions were statistically significant.

### Strategy and the relationships between perspective-taking and response-inhibition abilities

**Figure 3:**
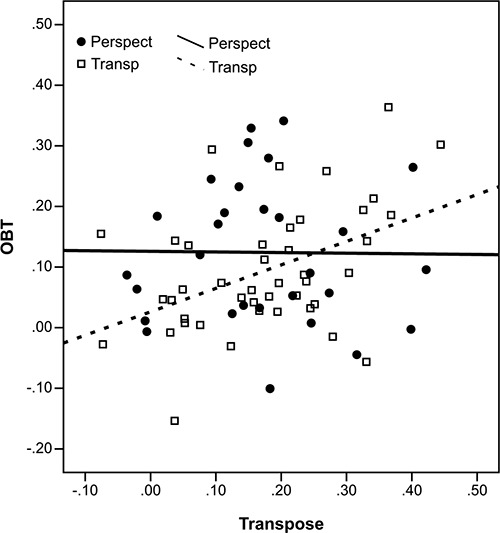
**Scatterplot of the association between the Composite RTs relating to the Transpose and OBT tasks**. Lines depict linear fit for subsamples defined by strategy reported (perspect = perspective transformers; transp = spatial transposers)

We examined whether self-reported strategy moderated the relationships between perspective-taking and response inhibition abilities by assessing correlations both within subgroups employing each type of strategy and collapsed across these subgroups, see Figure 3. When strategy was disregarded (*N* = 73), a positive relationship was found between performance on the OBT and Transpose tasks as measured by Composite RT, *r* = .245, *p* = .036. When the correlations were repeated within subgroups, the relationship between perspective-taking and response inhibition as measured by the OBT and Transpose tasks was found to be moderated by strategy. For the subgroup that reported having employed the disembodied spatial transposing strategy (*N* = 43), a highly significant positive correlation was found, *r* = .449, *p* = .003. Whereas, for the subgroup that reported having employed an embodied perspective transformation strategy (*N* = 29), there was no correlation between these tasks, *r* = −.011, *p* = .956. Nor were the tasks correlated when the 11 participants that reported having used both strategies were removed from the perspective transformation subgroup, *r* = −.065, *p* = .799, *N* = 18. The difference in correlation coefficients for perspective-transforming and transposing subgroups was statistically significant, *Z* = 1.96, *p* = .05 (Snedecor and Cochran, 1967).

## Discussion

The present study sought to clarify the cognitive processes involved in spatial perspective-taking by assessing whether strategy moderates the relationship between performance in tasks designed to measure perspective-taking (OBT) and response inhibition (Transpose). For the Transpose task, RTs were elevated for the incompatible relative to compatible condition, consistent with the costs of inhibiting a prepotent spatially compatible response in response to a cue (Gardner and Potts, 2011). For the OBT task, RTs were elevated for the front- relative to back-view condition, consistent with, depending upon putative route, either the costs of an embodied imagined transformation of perspective (e.g., Zacks et al., 1999; Blanke et al., 2005; Mohr et al., 2010), or the costs of inhibiting a spatially compatible response in response to the appearance of the front-view of the figure (Gardner and Potts, 2011, see also May and Wendt, 2012). The two strategy subgroups were not found to differ on overall speed of responding, or size of compatibility effect, in either the OBT or Transpose tasks. However, as predicted, participants’ self-report of which of these two strategies they had employed for the OBT task was found to moderate the relationship between the degree of elevation in response times resulting from incompatibility in the OBT and Transpose tasks. Specifically, response inhibition ability, indexed by the Transpose task, was found to be related to perspective-taking ability—but selectively for those reporting that they had adopted the disembodied spatial transposing strategy. This relationship was absent in those reporting having adopted an embodied perspective transformation strategy.

The selective association found between performance on the Transpose and OBT tasks implies that response inhibition ability predicts perspective-taking ability only among those that choose to take on another’s perspective using a “spatial transposing” strategy—that is, by reconfiguring spatial relationships as they appear from one’s own perspective. This association complements earlier work (Qureshi, 2008), by showing that the association between response inhibition and perspective-taking also generalizes to the perspective-taking performance measured by the OBT task, more specifically the relative ability to adopt a perspective differing from one’s own by 180° compared to 0°. The selective association is particularly important in providing evidence that this disembodied spatial transposing strategy is dissociable from an embodied perspective transformation strategy. Previously, the existence of this route was only implied by the absence of an association between trait empathy and perspective-taking ability otherwise present for those reporting having performed perspective transformations (Gronholm et al., 2012). Furthermore, the transposing subgroup also showed greater improvement in accuracy between the OBT and Transpose tasks. This could be explained in terms of their strategy for perspective-taking rendering the OBT task computationally equivalent to the Transpose task which leads to greater carry over from practice in comparison to the perspective transformation subgroup. These dissociations provide support for an independent disembodied route, consistent with findings for sensorimotor interference within the spatial updating literature for imaginal perspective changes in remembered environments (May, 2004).

Combined with earlier results (Gronholm et al., 2012), the present findings complete a double dissociation, implying that the spatial transposing and perspective transformation strategies reflect two separable routes to spatial perspective-taking. Where previously we described these strategies as empathic and non-empathic (Gronholm et al., 2012), we now suggest that this dissociation might be better characterized as between “embodied” perspective transformations and “disembodied” routes (Kessler and Rutherford, 2010; Stocker, 2012; Becchio et al., 2013; Tomasino and Rumiati, 2013). The embodied route, probably mediated by mental simulation of self motion (Lenggenhager et al., 2008; Steggemann et al., 2011), appears linked to social determinants such as trait empathy (Gronholm et al., 2012). The disembodied route, which the present results suggest involves the deliberate reconfiguration of spatial relationships as they appear from one’s own point of view, may be completely insensitive to social context or whether the new perspective is a position occupied by a person. This dissociation builds upon evidence suggesting dissociable processes for level 1 and level 2 perspective-taking (Michelon and Zacks, 2006; Kessler and Thomson, 2010), by implying that further fractionation is possible purely within the level 2 perspective-taking involved in the OBT task, confirming the hitherto untested hypotheses of other authors (e.g., Thakkar et al., 2009; Mohr et al., 2010; Thakkar and Park, 2010).

These results also inform debate on the suitability of the OBT and related tasks to measure spatial perspective-taking (Gardner and Potts, 2011; May and Wendt, 2012, under review). Given the way that it is operationalized with only four types of stimuli, the OBT task may be particularly susceptible to low-level alternative strategies. However, there are at least three reasons not simply to dismiss the OBT task as a test of spatial perspective-taking on the basis that it may be solved by the reconfiguration of spatial relationships as they appear from one’s own position. First, this issue does not appear to be unique to the OBT task. A similar mechanism could contribute to performance in other tasks employing laterality judgments (e.g., Michelon and Zacks, 2006; Kessler and Thomson, 2010, see May and Wendt, under review), although it is less likely to extend to tasks requiring participants to imagine the appearance of an array from a novel perspective (e.g., Langdon and Coltheart, 2001). Second, evidence that imitation also imposes a demand to inhibit incompatible S-R mappings (e.g., Ishikura and Inomata, 1995; Heyes and Ray, 2004; Jackson et al., 2006; Chiavarino et al., 2007), suggests that spatial transposing may be pervasive in face-to-face social interactions. Third, the present results imply that although the low-level reconfiguration of spatial relationships may contribute to performance in the OBT task, this may be restricted to a subset of participants adopting a particular “spatial transposing” strategy. Thus, this finding implies that identifying the interpersonal determinants of strategy selection may be a worthwhile avenue for research in spatial perspective-taking, and social interaction more generally (see Mohr et al., 2013).

The finding that the two dissociable perspective-taking processes may be reliably categorized by self-reported strategy also implies that the route to perspective-taking may be endogenously triggered. This evidence contrasts with other research showing exogenous triggering of embodied perspective-taking, either by revealing enhanced perspective-taking for body present compared with body absent conditions (Becchio et al., 2013), or by showing that congruence between the postures of the participant and an avatar facilitated performance (Kessler and Thomson, 2010). However, our finding that the presentation of a figure is not sufficient to elicit embodied perspective-taking corresponds to the finding that galvanic vestibular stimulation selectively disrupts mental task performance for participants adopting an egocentric rather than object-based transformation strategy (Lenggenhager et al., 2008). It also complements work demonstrating that the presence of a body was neither a necessary condition for response latency being related to the extent of imagined self rotation (Michelon and Zacks, 2006), nor for congruence effects between the participant’s body position and direction of imagined rotation (Kessler and Thomson, 2010). Although research to date implies that embodied perspective-taking can be both endogeneously and exogenously driven, the significance of the present results is that they demonstrate endogenous driven embodied perspective-taking for a task that, by inviting participants to step into the shoes of the schematic figure, might have been assumed likely to have triggered an embodied route exogenously. This corresponds with the view that the strategic modulation of embodied and disembodied routes is pervasive in various domains of cognition, including object based mental rotation, and language learning (Tomasino and Rumiati, 2013).

The absence of a correlation for the perspective transformation subgroup between perspective-taking and response inhibition, and the statistically significant difference in correlation coefficient compared to the spatial transformation subgroup, implies this executive function is not involved in the “embodied” route to perspective-taking to the same extent as for the “disembodied” route adopted by spatial transposers. This is consistent with earlier findings implying the fractionation of perspective-taking processes into cognitively efficient and cognitively demanding components (Michelon and Zacks, 2006; Samson et al., 2010; Qureshi et al., 2010), and with the different developmental trajectories of perspective-taking and executive function (Dumontheil et al., 2010). Previously, the cognitively demanding perspective-taking process was taken to be either the calculation of spatial relationships relative to an alternative viewpoint (Michelon and Zacks, 2006 —“level 2” knowledge; Flavell et al., 1981)or the selection of either the self or other perspective (Qureshi et al., 2010). Whereas, the cognitively efficient process in both studies was the calculation about what is visible from another viewpoint (level 1 knowledge). By contrast, in the present study, both routes involve the calculation of spatial relationships, but only the disembodied route appears to load onto response inhibition. This raises the possibility that a dedicated, domain specific, route also exists for level 2 perspective-taking, provided that one uses an embodied strategy (cf Amorim et al., 2006). We speculate that this route may place relatively light demands on domain general resources, although further research is required to assess this possibility.

Limitations of the correlational method adopted in the present study should be acknowledged. On one hand, our choice of the Transpose task as a measure of response inhibition could have elevated correlations in both subgroups due to shared variance attributable to procedural similarity between the OBT and Transpose tasks. On the other hand, methodological limitations could contribute to the absence of a statistically significant correlation for the embodied perspective transformation subgroup; the perspective transformation subgroup was smaller, and potentially more heterogeneous than the spatial transformation subgroup. Although the magnitude of the correlation coefficients reported here between perspective-taking and response inhibition should therefore be interpreted with caution, critically these correlations were found to differ significantly between subgroups. Differences in sample size would have reduced the power of this test, and shared variance attributable to task similarity would be expected to affect both subgroups equally. Nonetheless, our findings should ideally be corroborated using an experimental approach such as the dual task methodology used by Qureshi et al. (2010), despite contrasting selective associations being an established methodology for revealing evidence for dissociable processes (e.g., Asendorpf et al., 2002).

Finally, it should be noted that participants’ performance for the OBT and Transpose tasks did not show the close equivalence found in other research using the same tasks (Gardner and Potts, 2011). In the present study, overall performance was found to be better for the Transpose than the OBT task, both in terms of shorter RTs and fewer errors, and the size of the compatibility effect was greater in the Transpose task than in the OBT task. At first glance, these findings might be taken to imply that the Transpose task is not a good control for the OBT task, or, alternatively, that the disembodied spatial transposing route is more efficient than the embodied perspective transformation route. However, in the current experiment, all participants completed the Transpose task after the OBT task in order not to influence the strategy employed for the OBT task—a likely possibility had order been counterbalanced. Therefore, the between task differences in performance could be accounted for by practice in the first task (OBT) leading to better performance in a similar second task (Transpose), particularly for the compatible trials, and particularly for those adopting a disembodied spatial transposing strategy. Such differences were not found when different participants completed the two tasks (Gardner and Potts, 2011).

In conclusion, our main finding was a selective association whereby response inhibition was related to perspective-taking ability only among participants adopting a “spatial transposing” strategy—that is, by reconfiguring spatial relationships as they appear from one’s own perspective. Combined with earlier results (Gronholm et al., 2012), this evidence completes a double dissociation between two independent routes to perspective-taking in the OBT task. We propose that these routes either recruit “embodied” egocentric mental transformation processes, or involve the “disembodied” reconfiguration of spatial relationships. The contributions made by these findings are that they elucidate the processes involved in perspective-taking, imply that perspective-taking route is under higher order control, and lend support to the hypothesis that embodied routes to perspective-taking place minimal demands on domain general executive functions.

## Conflict of interest statement

The authors declare that the research was conducted in the absence of any commercial or financial relationships that could be construed as a potential conflict of interest.
